# Synergistic regulation of neuroinflammation by mitophagy and the cGAS–STING pathway following ischemic stroke

**DOI:** 10.3389/fnmol.2026.1990236

**Published:** 2026-10-26

**Authors:** Yu Xiao, Peijie Fu, Chun Zhao, Wenqi Song, Yutao Lu, Huiqin Wang, Xiaoping Yin, Zhiying Chen, Yuanyuan Xiang

**Affiliations:** 1 Department of Neurology, Clinical Medical College of Jiujiang University, Jiujiang, Jiangxi, China; 2 Jiujiang Clinical Precision Medicine Research Center, Jiujiang, Jiangxi, China; 3 Gastrointestinal Surgery Department, Third People's Hospital of Jiujiang City, Jiujiang, Jiangxi, China; 4 College of Stomatology, Jiujiang University, Jiujiang, Jiangxi, China; 5 Department of Plastic Surgery, Clinical Medical College of Jiujiang University, Jiujiang, Jiangxi, China

**Keywords:** cGAS–STING, crosstalk, ischemic stroke, mitophagy, neuroinflammation, synergy

## Abstract

Neuroinflammation is widely recognized as a major driver of secondary brain injury following ischemic stroke. Recent studies have shown that mitochondrial dysfunction-induced mitochondrial DNA (mtDNA) release is one of the key mechanisms activating the cyclic GMP-AMP synthase-stimulator of interferon genes (cGAS-STING) pathway. Mitophagy, as a quality control mechanism maintaining mitochondrial homeostasis, selectively clears damaged mitochondria, thereby limiting the leakage of abnormal mtDNA. Although previous reviews have outlined the general framework of the mitophagy-cGAS-STING interaction in neuroinflammation, this review places it within the specific context of ischemic stroke. It integrates the cell type-specificity of the neurovascular unit, explores both synergistic and interactive aspects, and evaluates targeted nanotherapeutic strategies. This article aims to explore the potential synergistic role of mitophagy and the cGAS-STING pathway in the pathological progression of ischemic stroke. First, the article elaborates on mitophagy and mitophagy-dependent regulation of neuroinflammation, highlighting its role in suppressing cGAS-STING activation by preventing mtDNA leakage. Second, it examines the cGAS-STING signaling pathway in neuroinflammation, with particular attention to cGAS-STING activation across neurovascular unit cell types (microglia, neurons, astrocytes, endothelial cells). Third, the functional synergy and potential molecular crosstalk between mitophagy and the cGAS–STING pathway in neuroinflammation are discussed. Finally, the article summarizes the potential therapeutic strategies targeting mitophagy and the cGAS-STING pathway, and further explores the emerging potential of microenvironment-responsive intelligent nanoplatforms to enhance drug delivery efficiency and multidimensional synergistic mechanisms by targeting the cGAS-STING pathway and mitochondria, thereby achieving precise immune regulation and improving therapeutic efficacy. This paper provides a mechanistic perspective on immune intervention for ischemic stroke and lays a theoretical foundation for future clinical translation strategies.

## 1 Introduction

Stroke is the second leading cause of death worldwide. Ischemic stroke accounts for more than 80% of all stroke cases and is a major cause of disability and mortality (Hilkens et al., 2024; Kleindorfer et al., 2021). In ischemic stroke, attenuation of neuroinflammation is considered an important step toward reducing brain tissue injury and improving functional outcomes (McCabe et al., 2026). Following ischemic stroke, cellular energy failure and ionic homeostasis imbalances lead to mitochondrial dysfunction and the release of damage-associated molecular patterns (DAMPs; Liu and Zhang, 2026). These molecules activate innate immune pathways in the central nervous system, triggering complex inflammatory cascades (Shi et al., 2019).

The cyclic GMP-AMP synthase (cGAS)-stimulator of interferon genes (STING) pathway, a cytosolic DNA-sensing innate immune pathway, has gained widespread attention in recent years for its ability to induce type I interferon production and initiate inflammatory responses (Paul et al., 2021). As a specialized form of autophagy, mitophagy maintains cellular homeostasis by eliminating dysfunctional mitochondria and participates in the regulation of neuroinflammation (Wu J. et al., 2025). Growing evidence indicates a close regulatory relationship between mitophagy and the cGAS–STING pathway after stroke (Zhou et al., 2024). After ischemic stroke, mitochondrial damage can lead to the leakage of mitochondrial DNA (mtDNA) and oxidized mitochondrial DNA (ox-mtDNA), which activate the cGAS–STING pathway and trigger downstream inflammatory cascades (Li et al., 2024; Zhao et al., 2021; Figure 1). Enhanced or impaired mitophagy can play an important regulatory role by reducing or increasing the accumulation of damaged mitochondria during cerebral ischemia–reperfusion injury (Huo et al., 2026; He et al., 2019; Yao et al., 2025). In addition, the cGAS–STING pathway may influence autophagy/mitophagy in return (Zhou et al., 2024). Therefore, elucidating the synergistic regulatory mechanisms between mitophagy and the cGAS–STING pathway in ischemic stroke may help deepen our understanding of post-stroke neuroinflammation and inform the exploration of novel therapeutic strategies.

**Figure 1:**
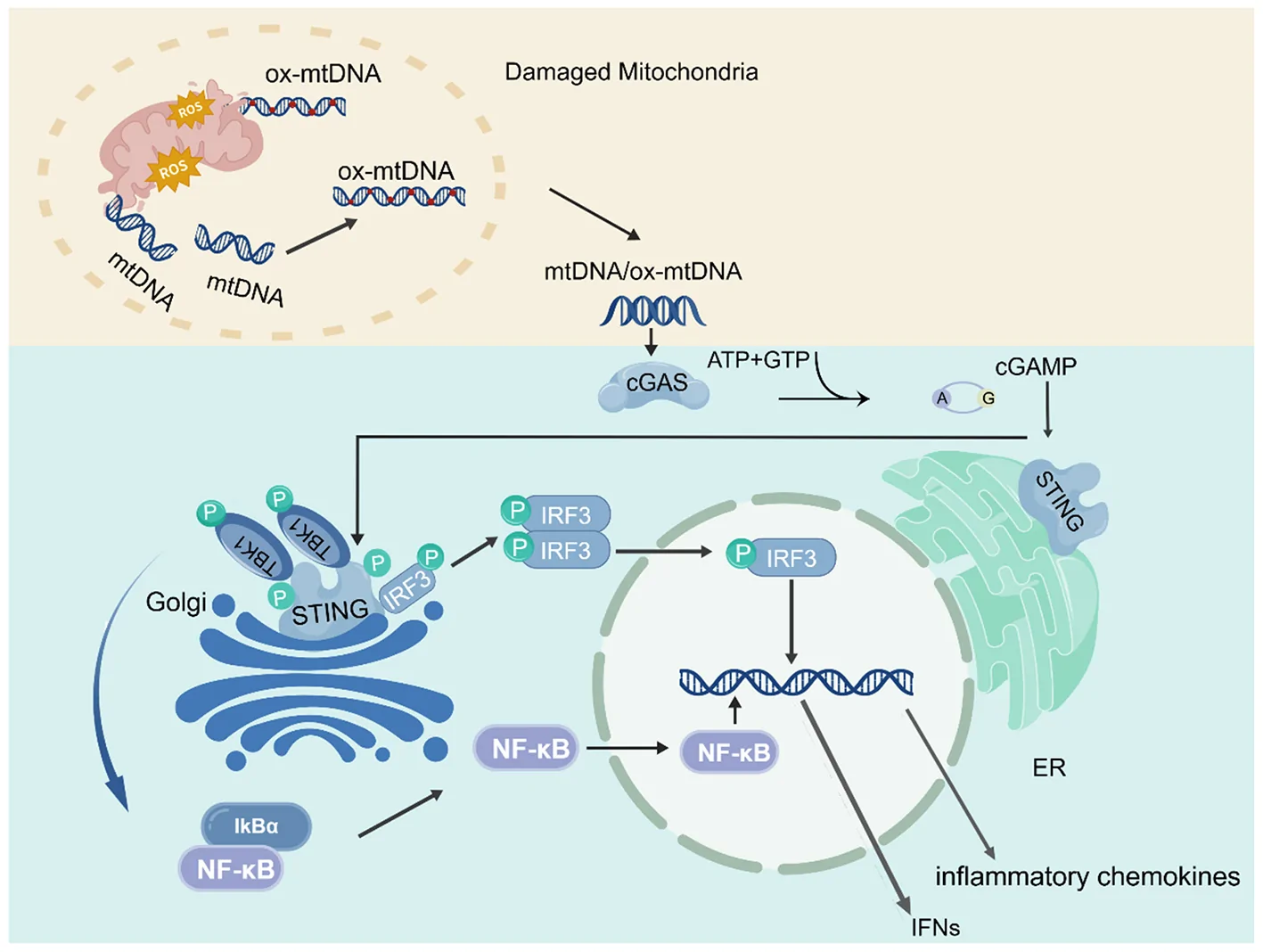
The cGAS–STING signaling cascade triggered by mitochondrial dysfunction. Following ischemic stroke, mitochondrial dysfunction in host cells leads to leakage of mtDNA and ox-mtDNA, thereby activating cGAS. cGAS then activates STING via the second messenger cGAMP. Activated STING exerts its effects through two pathways: it mediates the transcription and release of interferons (IFNs) via the IRF3 pathway; and it induces the transcription and release of inflammatory chemokines via the NF-κB pathway. Created with BioGDP.com (Jiang et al., 2025).

Overall, mitophagy and the cGAS–STING pathway not only exert independent regulatory effects in neuroinflammation but also exhibit coordinated regulation across multiple aspects. Both processes contribute to key pathophysiological events in neuroinflammation, including microglial activation and the release of inflammatory mediators. This review summarizes current evidence on how mitophagy and the cGAS–STING signaling pathway independently and synergistically regulate post-stroke neuroinflammation, with emphasis on their crosstalk. It further discusses potential therapeutic strategies targeting both pathways, with the aim of informing future treatment development.

## 2 The critical role of mitophagy in ischemic stroke

### 2.1 Mitophagy pathways

Mitophagy exhibits basal activity under physiological conditions, contributing to mitochondrial quality control and maintaining cellular homeostasis and basic metabolic needs (Kelly et al., 2024; Wang et al., 2023). In ischemic stroke, mitochondrial dysfunction further activates mitophagy (Qin et al., 2022). The mechanisms of mitophagy are mainly classified into two categories: ubiquitin-dependent pathways and receptor-dependent pathways.

Under physiological conditions, PTEN-induced kinase 1 (PINK1) is imported into the mitochondria through the translocase complexes of the outer and inner mitochondrial membranes (TOM/TIM23) and is rapidly degraded by proteases such as mitochondrial processing peptidase (MPP) and presenilin-associated rhomboid-like protease (PARL; Jin et al., 2010; Greene et al., 2012). When mitochondrial damage occurs, and mitochondrial membrane potential (MMP) decreases, PINK1 is unable to enter the inner mitochondrial membrane and instead accumulates on the outer mitochondrial membrane (Jin et al., 2010). Accumulated PINK1 undergoes dimerization and autophosphorylation, leading to its activation (Rasool et al., 2022; Gan et al., 2022). Activated PINK1 phosphorylates Ser65 on ubiquitin chains on outer mitochondrial membrane proteins, generating phosphorylated ubiquitin (pUb; Koyano et al., 2014; Kazlauskaite et al., 2014). pUb, as a signaling molecule, recruits the cytosolic E3 ubiquitin ligase Parkin, which is further activated by PINK1 (Kane et al., 2014). Activated Parkin ubiquitinates various outer mitochondrial membrane proteins, and the ubiquitin chains are subsequently recognized by autophagy receptors. These receptors interact with microtubule-associated protein 1 light chain 3 (LC3) on autophagosomes via LC3-interacting regions (LC3R), thereby facilitating the sequestration of damaged mitochondria into autophagosomes for degradation (Narendra et al., 2010). In oxygen–glucose deprivation/reperfusion (OGD/R) and middle cerebral artery occlusion (MCAO) models, the expression of PINK1, Parkin, and LC3-related markers is significantly increased, indicating activation of the PINK1–Parkin pathway under cerebral ischemia/reperfusion conditions (Zhao et al., 2025; Shang et al., 2025; Zhong et al., 2024).

In parallel to this ubiquitin-dependent clearance, receptor-mediated mitophagy serves as another critical defense mechanism against stroke-induced hypoxia. Notably, BNIP3 and its homolog BNIP3L/Nix are key regulators of receptor-dependent mitophagy. CI/RI enhances the expression of hypoxia-inducible factor-1α (HIF-1α), promoting the transcription of the downstream genes BNIP3 and NIX (Guo, 2017; Bellot et al., 2009). Both BNIP3 and NIX contain LIR motifs and transmembrane domains, thereby mediating the interaction between damaged mitochondria and autophagosomes (Yamashita et al., 2025). The absence of BNIP3L inhibits ischemia/reperfusion-induced mitophagy and exacerbates tissue injury, whereas restoring its expression reactivates mitophagy and reduces brain damage (Yuan et al., 2017; Chen et al., 2020). These findings suggest that the BNIP3/Nix pathway plays a critical role in regulating mitophagy under hypoxic conditions and contributes significantly to mitigating ischemic brain injury. FUNDC1 is a triple-transmembrane protein located on the outer mitochondrial membrane (OMM), whose N-terminal cytosolic domain contains a typical LIR that directly binds LC3, thereby mediating the fusion of damaged mitochondria with autophagosomes (Kuang et al., 2016; Liu et al., 2012). Under normoxic conditions, Ser13 and Tyr18 of FUNDC1 are phosphorylated by casein kinase-2 (CK2) and Src kinase, respectively, thereby inhibiting its interaction with LC3 and suppressing mitophagy (Kuang et al., 2016; Chen et al., 2016). During cerebral ischemia and hypoxia, the mitochondrial serine/threonine protein phosphatase PGAM5 dephosphorylates Tyr18 and Ser13 of FUNDC1, thereby dissociating CK2 and Src from FUNDC1, enhancing FUNDC1 binding to LC3 and promoting mitophagy (Liu et al., 2012; Chen et al., 2014; Lv et al., 2017).

These signaling pathways have expanded our understanding of the regulatory network of mitophagy. Further elucidation of their complexity and key regulatory targets will help develop more precise targeted intervention strategies, providing more effective and specific therapeutic options for ischemic stroke.

### 2.2 Dual role of mitophagy in neuroinflammation after stroke

Following ischemic stroke, the body rapidly initiates an immune-inflammation response to clear necrotic cells and promote repair. However, when this inflammatory response becomes uncontrolled, the release of pro-inflammatory cytokines and reactive oxygen species (ROS) exacerbates cell death and disrupts the blood-brain barrier (BBB; Wang M. et al., 2024; Yang et al., 2023). Recent studies have indicated that mitophagy, an essential mechanism for maintaining cellular and mitochondrial homeostasis, plays a critical role in regulating neuroinflammation after stroke, exhibiting distinct temporal and intensity dependence. Mitophagy exerts neuroprotective effects by selectively removing damaged mitochondria. During cerebral ischemia/reperfusion, mitochondrial damage leads to the leakage of mtDNA, including oxidized mtDNA (ox-mtDNA), which activates the cGAS–STING pathway and triggers neuroinflammatory responses (Li et al., 2024; Guo et al., 2026; Huang F. Q. et al., 2025). Blocking mtDNA has been shown to attenuate cGAS–STING-mediated neuroinflammation after stroke and reduce brain injury (Guo et al., 2026; Huang F. Q. et al., 2025). Notably, studies directly examining whether mtDNA-mediated regulation of mitophagy affects the cGAS–STING pathway in ischemic stroke models remain limited, although this mechanism has been shown to ameliorate inflammatory responses in ischemia/reperfusion injury in other organs (Zhou et al., 2026; Li Y. et al., 2026). Specifically, activation of the HIF-1α/BNIP3 pathway or enhancement of Parkin-dependent mitophagy during ischemia/reperfusion injury has been shown to directly downregulate TLR9 and NLRP3 inflammasome activity, thereby alleviating neuronal injury (Huo et al., 2026; He et al., 2019; Guo et al., 2026; Huang F. Q. et al., 2025). Meanwhile, PINK1/Parkin-mediated mitophagy significantly reduces microglial pyroptosis and alleviates neuronal injury (Yao et al., 2025). Notably, mitophagy may exacerbate inflammatory responses during cerebral ischemia. A recent study showed that, in an *in vitro* oxygen–glucose deprivation (OGD) model, microglial engulfment of apoptotic neurons induced upregulation of dynamin-related protein 1 (Drp1), triggering excessive mitochondrial fission and mitophagy, which resulted in an energy deficit in microglia, subsequently impaired their phagocytic capacity, and exacerbated neuroinflammation; inhibition of mitophagy alleviated the inflammatory response (Wang et al., 2026). Another study found that CKLF/CKLF1 (chemokine-like factor 1) increased the number of autophagosomes during mitophagy while impairing lysosomal function, including mitophagosome–lysosome fusion, lysosomal biogenesis, and acidification (Wang H. et al., 2024). This study indicated that even when the number of autophagosomes increases, an incomplete mitophagic flux can ultimately lead to the accumulation of damaged mitochondria in microglia, thereby excessively activating microglia and inducing inflammation; pharmacological blockade or genetic silencing of mitophagy suppressed this process (Wang H. et al., 2024).

Current evidence does not yet provide a clear signaling threshold or specific context for distinguishing the beneficial from the detrimental effects of mitophagy on neuroinflammation after ischemic stroke. Existing studies instead suggest that the extent of mitophagy and the integrity of mitophagic flux may jointly determine its ultimate biological effects (Wang et al., 2026; Wang H. et al., 2024). This potential double-edged effect of mitophagy may inform the development of therapeutic strategies for post-stroke neuroinflammation. Therapeutic interventions should therefore not simply aim to promote or inhibit mitophagy; rather, they should aim to restore effective mitophagic flux and re-establish mitochondrial homeostasis. For example, enhancing mitophagy may be beneficial when damaged mitochondria accumulate and mitophagic capacity is insufficient, whereas inhibiting excessive mitophagy may be more appropriate when sustained mitophagy leads to cellular energy depletion.

In summary, mitophagy may exert dual effects on neuroinflammation after stroke. This dual effect may not simply depend on whether mitophagy is “enhanced” or “inhibited,” but rather on the dynamic balance among the extent of mitophagy, cellular energy requirements, and the integrity of mitophagic flux.

## 3 The role of cGAS–STING in neuroinflammation after stroke

### 3.1 Activation of cGAS–STING

In vertebrate cells, the cGAS–STING pathway is a critical innate immune mechanism for recognizing abnormal cytosolic double-stranded DNA (dsDNA; Zhang et al., 2022). Following ischemic stroke, the cytosolic dsDNA that activates cGAS primarily originates from leaked mitochondrial DNA (mtDNA) and oxidized mtDNA (ox-mtDNA; Li et al., 2024; Kong et al., 2022). Among these, mtDNA, due to its retention of unmethylated CpG motifs similar to those in bacterial DNA, exhibits strong immunogenicity and is more likely to activate the cGAS–STING pathway (Riley and Tait, 2020). Therefore, mtDNA leakage resulting from mitochondrial dysfunction after stroke may be an important contributor to the abnormal activation of cGAS–STING. It is also worth noting that neutrophil extracellular traps (NETs), which contain large amounts of DNA from neutrophil chromatin, can be phagocytosed and enter the cellular environment, thereby activating cGAS (Apel et al., 2021). Recent studies have shown that inhibiting NET release can alleviate the inflammatory response triggered by cGAS–STING activation and promote neuronal survival (Wang et al., 2021; Sun et al., 2023).

cGAS plays a crucial role in sensing abnormal DNA in the cytosol and activating downstream signaling cascades. This pathway is essential in the onset and amplification of neuroinflammation after ischemic stroke, reflecting the cell's fine immune regulation of damage-associated signals.

### 3.2 Downstream signaling pathways of cGAS–STING

After recognizing cytosolic double-stranded DNA (dsDNA), cGAS undergoes a conformational change and catalyzes the synthesis of the second messenger 2′3′-cGAMP from ATP and GTP through non-canonical 2′-5′ and 3′-5′ phosphodiester bonds. Subsequently, cGAMP binds to STING on the endoplasmic reticulum (ER) membrane (Wu et al., 2013; Sun et al., 2013; Wu S. et al., 2024; Du and Chen, 2018). In human cells and mouse embryonic fibroblasts, damaged cells can directly transfer cGAMP to neighboring cells via gap junctions (GJs; Ablasser et al., 2013b). STING is a ~42 kDa protein containing four transmembrane domains. Upon cGAMP binding, STING undergoes dimerization and oligomerization, accompanied by conformational rearrangement (Wu S. et al., 2024; Ablasser et al., 2013a). Activated STING is exported from the ER via ER exit sites (ERES) and budded off in COPII-coated vesicles (dependent on proteins such as Sar1 and Sec23/24). It is then transported to the ER–Golgi intermediate compartment (ERGIC) or the Golgi apparatus (Jeltema et al., 2023). In the Golgi apparatus, STING recruits and binds TANK-binding kinase 1 (TBK1), leading to a reciprocal phosphorylation that amplifies the activation signal (Tanaka and Chen, 2012). A membrane-proximal cysteine residue of STING undergoes palmitoylation, which stabilizes and sustains the signaling complex; inhibition of palmitoylation or mutation of this cysteine residue significantly reduces the type I interferon (IFN-I) response (Mukai et al., 2016). Activated TBK1 phosphorylates IRF3 using STING as a scaffold. Phosphorylated IRF3 then dimerizes and translocates into the nucleus to induce IFN transcription (Tanaka and Chen, 2012; Liu et al., 2015). Meanwhile, STING can also recruit mitogen-activated protein kinase kinase kinase 14 (MAP3K14/NIK) and the IκB kinase (IKK) complex, thereby activating the NF-κB pathway and promoting the transcription of inflammatory chemokines such as IL-6 and TNF-α (Liu et al., 2025).

The cGAS–STING pathway functions as a “signal amplifier,” linking mitochondrial homeostasis imbalance to inflammatory responses by sensing cytosolic DNA abnormalities. In the complex microenvironment of ischemic stroke, this transition from sensing to response highlights the adaptability of the innate immune system and further deepens our understanding of immune system complexity in maintaining cellular integrity.

### 3.3 cGAS–STING-mediated neuroinflammation in the neurovascular unit (NVU)

In cGAS–STING-mediated neuroinflammation, microglia emerge as a major contributor. Studies have shown that following ischemic stroke, activation of the cGAS–STING pathway influences microglial polarization states. In cerebral ischemia/reperfusion models, activation of cGAS–STING has been associated with shifts toward a pro-inflammatory phenotype (Kong et al., 2022; Jiang et al., 2021). In addition, a functional link exists between the cGAS–STING pathway and inflammasomes. Overactivation of cGAS–STING exacerbates NLRP3 and AIM2 inflammasome-mediated inflammatory responses and microglial pyroptosis, whereas pharmacological inhibition can reverse these detrimental effects (Zhang et al., 2025; Li Q. et al., 2026; Li et al., 2020). Meanwhile, the STING inhibitor H151 has been shown to reverse microglia-mediated synaptic engulfment of neurons, suggesting a potential regulatory role for STING in post-stroke microglial phagocytic activity (Wu C. et al., 2024). Notably, modulation of cGAS–STING signaling may also facilitate remyelination and long-term recovery after stroke, indicating that its effects may be temporally dependent (Maimaiti et al., 2024).

Importantly, microglial activation is strictly regulated by upstream epigenetic mechanisms. For instance, epigenetic regulation, including HDAC3 transcriptional axis activity and m6A RNA methylation, also participates in the transcriptional control of cGAS–STING signaling in microglia. Through the IFN-dependent pathway, inflammatory mediators such as IL-6 and IFN-β are released, driving microglial polarization and NLRP3 inflammasome activation, ultimately contributing to neuroinflammatory injury (Liao et al., 2020; Li Y. et al., 2023). Beyond its canonical interaction with STING, cGAMP can also activate alternative downstream pathways with protective effects. Recent studies have demonstrated that cGAMP enhances microglial/macrophage phagocytic activity in ischemic stroke via activation of the TREM2–DAP10–PI3K pathway (Li X. et al., 2026). Moreover, cGAMP has been shown to alleviate oxidative stress and ferroptosis in ischemic stroke by activating the Nrf2/HO-1/GPX4 signaling axis (Li et al., 2025). These findings highlight the complexity of the cGAS–STING signaling network. On one hand, moderate activation of cGAS–STING facilitates clearance of necrotic debris after stroke; on the other hand, excessive activation drives robust inflammatory responses that aggravate brain injury.

In addition to microglia, the cGAS–STING pathway is also present in other key components of the neurovascular unit (NVU), including neurons, astrocytes, and brain microvascular endothelial cells (bECs), exhibiting a degree of cell-type specificity (Figure 2). Although neurons have traditionally been regarded as passive targets of inflammation, recent studies suggest that they possess active intrinsic immune mechanisms. Li et al. found that oxidative stress following cerebral ischemia/reperfusion induces ox-mtDNA formation in neurons, thereby activating the cGAS–STING pathway in both neurons and microglia. Specifically, cGAS–STING activation appears to occur first in neurons and subsequently in microglia, suggesting a temporal sequence of activation (Li et al., 2024). Huang J. et al. (2025) further demonstrated that inhibition of neuronal cGAS–STING signaling enhances the expression of proteins associated with neuroplasticity and angiogenesis, while reducing neuronal apoptosis and inflammatory responses. In astrocytes following ischemia, Lei et al. (2026) reported a significant downregulation of SIRT3, which removes its inhibitory effect on cGAS–STING signaling. Restoration of SIRT3 activity effectively suppresses the cGAS–STING pathway, reduces pro-inflammatory cytokine release, and significantly improves neurological function. Brain microvascular endothelial cells (bECs) form the anatomical basis of the blood-brain barrier, and ischemia-induced endothelial dysfunction often precedes peripheral immune cell infiltration into the central nervous system (Göttert et al., 2026). Göttert et al. found that ischemia activates the mechanotransduction regulators YAP/TAZ in endothelial cells. Conditional deletion of YAP/TAZ in endothelial cells suppresses the release of the downstream chemokine CXCL10 from the cGAS–STING pathway. In addition, such deletion promotes the recruitment of certain myeloid cells while inhibiting microglial activation, ultimately contributing to the formation of an anti-inflammatory vascular microenvironment and significantly reducing chronic infarct volume (Göttert et al., 2026). This finding extends the regulatory network of cGAS–STING to endothelial dysfunction and vascular microenvironment remodeling.

**Figure 2:**
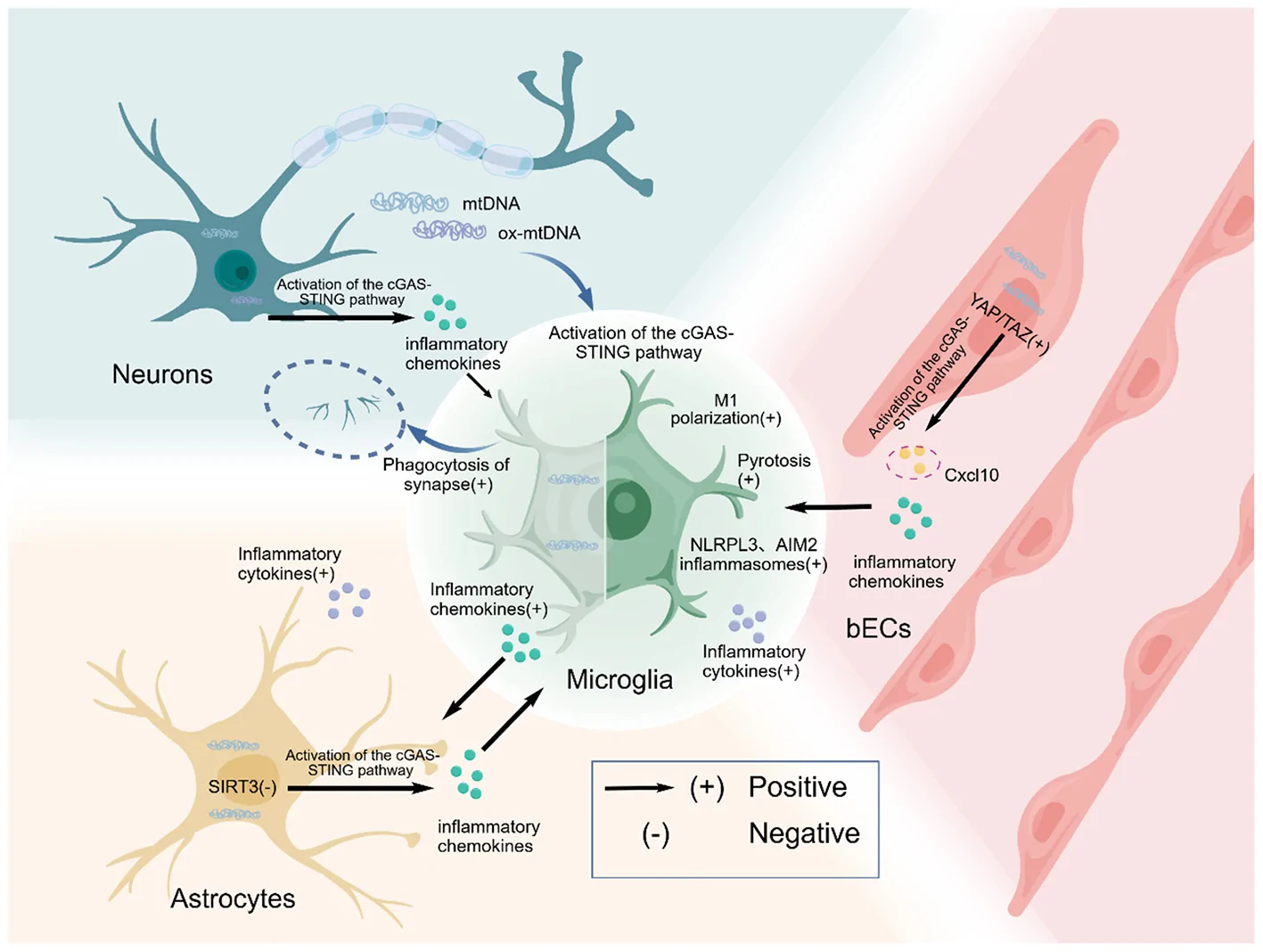
cGAS–STING-mediated neuroinflammatory crosstalk within the NVU after ischemic stroke. Microglia: impaired mitophagy causes mtDNA leakage, activating cGAS–STING to drive M1 polarization, inflammasome activation (NLRP3/AIM2), and pyroptosis. Neurons: oxidative stress-induced ox-mtDNA activates cGAS–STING in both neurons and adjacent microglia. Astrocytes: ischemia-induced SIRT3 downregulation and mtDNA leakage trigger cGAS–STING-mediated chemokine release. bECs: mitochondrial dysfunction and YAP/TAZ activation synergistically promote CXCL10 production. Ultimately, the pro-inflammatory factors released from these diverse cellular sources converge on glial cells, forming a potential positive feedback loop that may exacerbate neuroinflammation. Created with BioGDP.com (Jiang et al., 2025).

The components of the NVU do not act independently in activating cGAS–STING signaling, and coordinated cGAS–STING signaling may also involve intercellular cGAMP transfer. Studies have demonstrated that, in human cells and mouse embryonic fibroblasts, damaged cells can directly transfer cGAMP to neighboring cells through gap junctions (GJs), thereby inducing STING-dependent immune responses in recipient cells (Ablasser et al., 2013b). Observations from neuron–microglia co-culture systems further support this mechanism, as GJ blockade significantly suppressed cGAS/STING activation (Elzinga et al., 2022). Such intercellular transfer may amplify inflammatory signaling by extending cGAS–STING activation from initially injured cells to neighboring NVU components. These findings raise the possibility that targeting cGAS–STING in a single NVU cell type may not fully interrupt intercellular inflammatory signaling, and that combinatorial strategies targeting cell-intrinsic pathway activation and intercellular signal transmission may warrant further investigation.

These studies indicate that the cGAS–STING pathway and its downstream signaling integrate multiple components of the neurovascular unit into a coordinated regulatory network, providing new insights into the regulation of the post-stroke immune microenvironment and therapeutic targeting. However, the cell-type-specific roles and mechanisms of cGAS–STING signaling within different NVU components after stroke, as well as the interactions among these components, still require further investigation.

## 4 Stroke neuroinflammation: functional synergy and molecular crosstalk between mitophagy and the cGAS–STING pathway

### 4.1 Functional synergy in the regulation of post-stroke neuroinflammation

In ischemic stroke, mitochondrial damage represents a common and pivotal initiating factor for both mitophagy induction and cGAS–STING activation. The brain relies predominantly on aerobic glucose metabolism for energy production and possesses minimal energy reserves, rendering it highly vulnerable to ischemia and hypoxia. Given the brain's high energy demand, mitochondria play a critical role in generating ATP through the respiratory chain and oxidative phosphorylation, thereby maintaining mitochondrial integrity and ensuring a stable energy supply (Zhang et al., 2024). During ischemic stroke, hypoxia and ischemia impair mitochondrial ATP production, while persistent opening of ion channels leads to Ca^2+^ influx, activation of lipases and proteases, and dysfunction of the mitochondrial respiratory chain. This results in excessive generation of reactive oxygen species (ROS), ultimately causing mitochondrial structural damage and functional impairment (Sims and Muyderman, 2010). These pathophysiological events can lead to a decline in MMP, which in turn activates outer mitochondrial membrane (OMM)-localized mitophagy receptors (e.g., BNIP3, NIX, and FUNDC1), thereby initiating mitophagy. Through the selective removal of dysfunctional mitochondria, mitophagy maintains mitochondrial quantity, structural integrity, and functional homeostasis, thus preserving intracellular stability. As described above, to some extent, mitophagy may exert anti-inflammatory and protective effects by suppressing the mtDNA–cGAS–STING axis, downregulating the NLRP3 inflammasome, and reducing microglial pyroptosis (Huo et al., 2026; He et al., 2019; Yao et al., 2025; Zhang T. et al., 2026; Zhang C. et al., 2026). In contrast, the cGAS–STING pathway functions as a cytosolic DNA sensor and inflammatory signaling system that converts aberrant DNA signals into downstream innate immune responses, including TBK1–IRF3/NF-κB-mediated production of type I interferons and inflammatory mediators, inflammasome activation, and promotion of the M1 phenotypic transition of microglia; inhibition of this pathway can also exert anti-inflammatory effects (Kong et al., 2022; Jiang et al., 2021; Zhang et al., 2025; Li Q. et al., 2026; Li et al., 2020; Liao et al., 2020). Therefore, their regulation may produce functional synergy in post-stroke neuroinflammation, closely linking upstream mitochondrial quality control with downstream inflammatory mechanisms.

### 4.2 Molecular crosstalk between mitophagy and the cGAS–STING pathway

#### 4.2.1 mtDNA is the most direct molecular bridge linking mitophagy and cGAS–STING

Mitochondrial DNA (mtDNA) can enter the cytosol through multiple mechanisms, primarily via permeabilization of the mitochondrial outer membrane by BAX/BAK pores and opening of the mitochondrial permeability transition pore (mPTP; McArthur et al., 2018; Xian et al., 2022; Liu et al., 2022). Free cytosolic mtDNA serves as a molecular link connecting mitochondrial damage and innate immunity. Upon sensing cytosolic mtDNA, the cGAS–STING pathway is activated, producing the second messenger cGAMP, which subsequently activates STING (Wu et al., 2013; Sun et al., 2013). Activation of the cGAS–STING pathway ultimately leads to transcriptional upregulation of type I interferons and pro-inflammatory cytokines, promoting microglial activation and inducing the release of inflammatory mediators, including IL-1β, TNF-α, and IL-6, as well as chemokines such as CXCL10. These factors not only trigger acute inflammatory responses but also activate neighboring glial cells and modulate neuronal function, thereby predisposing the brain to chronic neuroinflammation (Zhou et al., 2024; Göttert et al., 2026). Therefore, the release of mtDNA not only highlights a critical transition from mitochondrial injury to immune activation after ischemic stroke, but also provides a mechanistic basis for understanding how mitophagy may suppress excessive cGAS–STING activation by limiting mtDNA release.

#### 4.2.2 Effects of cGAS–STING on the autophagy and lysosomal systems

The relationship between cGAS–STING signaling and the autophagy–lysosomal system provides a mechanistic framework for considering its potential relationship with mitophagy. Following STING activation, STING traffics from the endoplasmic reticulum to the ER–Golgi intermediate compartment (ERGIC), where it forms a membrane platform that promotes LC3 lipidation and facilitates a noncanonical form of autophagy through WIPI2, ATG5, and other factors (Gui et al., 2019; Wan et al., 2023). In a study based on MCAO/OGD-R models, STING directly bound LC3 through its TM domain to activate autophagy, thereby promoting a pro-inflammatory phenotype in microglia (Kong et al., 2024). Another study found that cGAS–STING activation was associated with excessive autophagy in neurons during ischemia/reperfusion (Fuyun et al., 2025). In addition, during cerebral ischemia/reperfusion, cGAS–STING can regulate NCOA4-mediated ferritinophagy, suggesting that this pathway not only affects autophagy in general but may also participate in certain forms of selective autophagy (Wang et al., 2025; Li B. et al., 2023). Fusion between lysosomes and autophagosomes is a downstream step in mitophagy, and whether mitophagy ultimately exerts protective effects depends not only on autophagosome formation but also on the successful fusion of autophagosomes with lysosomes, as well as lysosomal biogenesis and acidification (Wang H. et al., 2024; Sui et al., 2024; Ke, 2024). Studies of cerebral ischemia/reperfusion have shown that inhibition of cGAS–STING promotes autophagosome–lysosome fusion mediated by the interaction of the autophagosomal outer-membrane SNARE proteins STX17 and SNAP29 with VAMP8, thereby restoring autophagic flux (Zhang et al., 2025). In addition, STING is also linked to lysosomal biogenesis and homeostasis. Specifically, STING activation can promote TFEB (transcription factor EB) nuclear translocation through GABARAP lipidation, thereby promoting lysosomal biogenesis; STING-induced autophagy induces ALIX-mediated ESCRT machinery recruitment to mitigate endolysosomal perturbation (Huang T. et al., 2025; Lv et al., 2024). These studies suggest that cGAS–STING may be mechanistically linked to the autophagy and lysosomal systems. These findings should, however, be distinguished from evidence specifically demonstrating mitophagy.

#### 4.2.3 The STING–TBK1–OPTN axis: an emerging mechanism linking cGAS–STING to selective mitophagy

Recent studies have shown that mitochondrial damage can promote the recruitment of STING to damaged mitochondria and activate TBK1; TBK1 then phosphorylates the autophagy receptor OPTN, enhancing OPTN recognition and recruitment to damaged mitochondria and further promoting PINK1–Parkin-dependent mitophagy (Huang et al., 2026a,b). This mechanism has been reported independently in recent studies, one of which clearly demonstrated that STING1, following recruitment to damaged mitochondria, activates TBK1 and promotes OPTN Ser177 phosphorylation and PINK1–PRKN-dependent mitophagy (Huang et al., 2026a). Another study similarly identified the STING–OPTN axis as an upstream regulatory mechanism of TBK1-dependent mitophagy (Huang et al., 2026b). This mechanism suggests that STING may function not only as a “downstream inflammatory sensor” induced by mitochondrial damage but also participate in feedback regulation of damaged mitochondrial clearance. In other words, STING–TBK1–OPTN may constitute another common node linking innate immune sensing with mitochondrial quality control and provide a potential molecular basis for their bidirectional regulation.

However, current studies are derived mainly from mechanistic cellular models, and direct validation in ischemic stroke models remains insufficient. Therefore, this pathway is currently more appropriately regarded as a potential mechanism rather than an adequately established stroke-specific mechanism. Future studies should further validate the actual role of this axis after stroke using ischemic stroke-specific models and cell type-specific interventions.

#### 4.2.4 Cell type- and time-specificity of the crosstalk

The interaction between cGAS–STING and autophagy within the neurovascular unit (NVU) exhibits a certain degree of cell type dependence. During cerebral ischemia/reperfusion, STING directly binds LC3 through its TM domain to activate autophagy, promoting a pro-inflammatory phenotype in microglia and exacerbating post-stroke neuroinflammation (Kong et al., 2024). Another study showed that cGAS–STING activation was associated with excessive autophagy and injury in neurons during cerebral ischemia/reperfusion, whereas inhibition of cGAS–STING alleviated this injury (Fuyun et al., 2025). In contrast, astrocytes and brain microvascular endothelial cells currently appear to be more prominently involved in cGAS–STING-mediated inflammatory signaling, whereas evidence directly linking STING activation to mitophagy remains very limited. Overall, this cell type-specific crosstalk within the NVU is primarily centered on general autophagy rather than mitophagy and should not be simply extrapolated to all cell types within the NVU.

In a rat tMCAO model, the key molecules of the cGAS–STING pathway (cGAS, STING, IRF3, TBK1, and P65) and their phosphorylation levels were all upregulated in a time-dependent manner within 24 h after cerebral ischemia/reperfusion injury. STING was significantly increased in neurons as early as 4 h, whereas its expression gradually increased in microglia and approached maximal levels at 12–24 h, indicating that the cGAS–STING pathway was activated first in neurons and subsequently increased in microglia after cerebral ischemia/reperfusion injury (Li et al., 2024). During the acute phase of ischemic stroke (0–24 h), moderate activation of PINK1/Parkin-mediated mitophagy enhances the clearance of damaged mitochondria and alleviates neuroinflammation; in contrast, during the subacute and chronic phases, sustained autophagy, particularly that driven by STING or endoplasmic reticulum stress–CHOP signaling, is associated with autophagic cell death, phagoptosis, and persistent neuroinflammation (Wu J. et al., 2025). However, studies examining the temporal dynamics and dependence of the crosstalk between cGAS–STING and mitophagy remain limited, and the precise regulatory relationship between the two at different stages requires further clarification.

### 4.3 Evidence for bidirectional regulation between cGAS–STING and mitophagy: current status and limitations

Existing studies suggest that mitophagy and cGAS–STING may be bidirectionally linked. In ischemic stroke, the mtDNA–cGAS–STING axis is relatively well established, with mtDNA activating the cGAS–STING–TBK1–IRF3/NF-κB inflammatory signaling pathway. Enhanced mitophagy may participate in suppressing this inflammatory pathway (Li et al., 2024; Guo et al., 2026; Huang F. Q. et al., 2025). In MCAO/OGD-R models, STING has been shown to regulate microglial polarization through interaction with LC3 during autophagy and to be associated with excessive autophagy in neurons during ischemia/reperfusion (Kong et al., 2024; Fuyun et al., 2025). However, these findings mainly concern general autophagy and cannot be equated with direct regulation of specific mitophagy by STING. Recent studies have further shown that STING can promote selective clearance of damaged mitochondria through TBK1–OPTN, providing direct molecular evidence for STING-mediated regulation of PINK1/PRKN-dependent mitophagy, but this mechanism has not yet been validated in ischemic stroke models (Huang et al., 2026a). Therefore, the two are currently more appropriately described as exhibiting functional synergy and potential molecular crosstalk rather than as constituting a fully established bidirectional regulatory loop (Figure 3).

**Figure 3:**
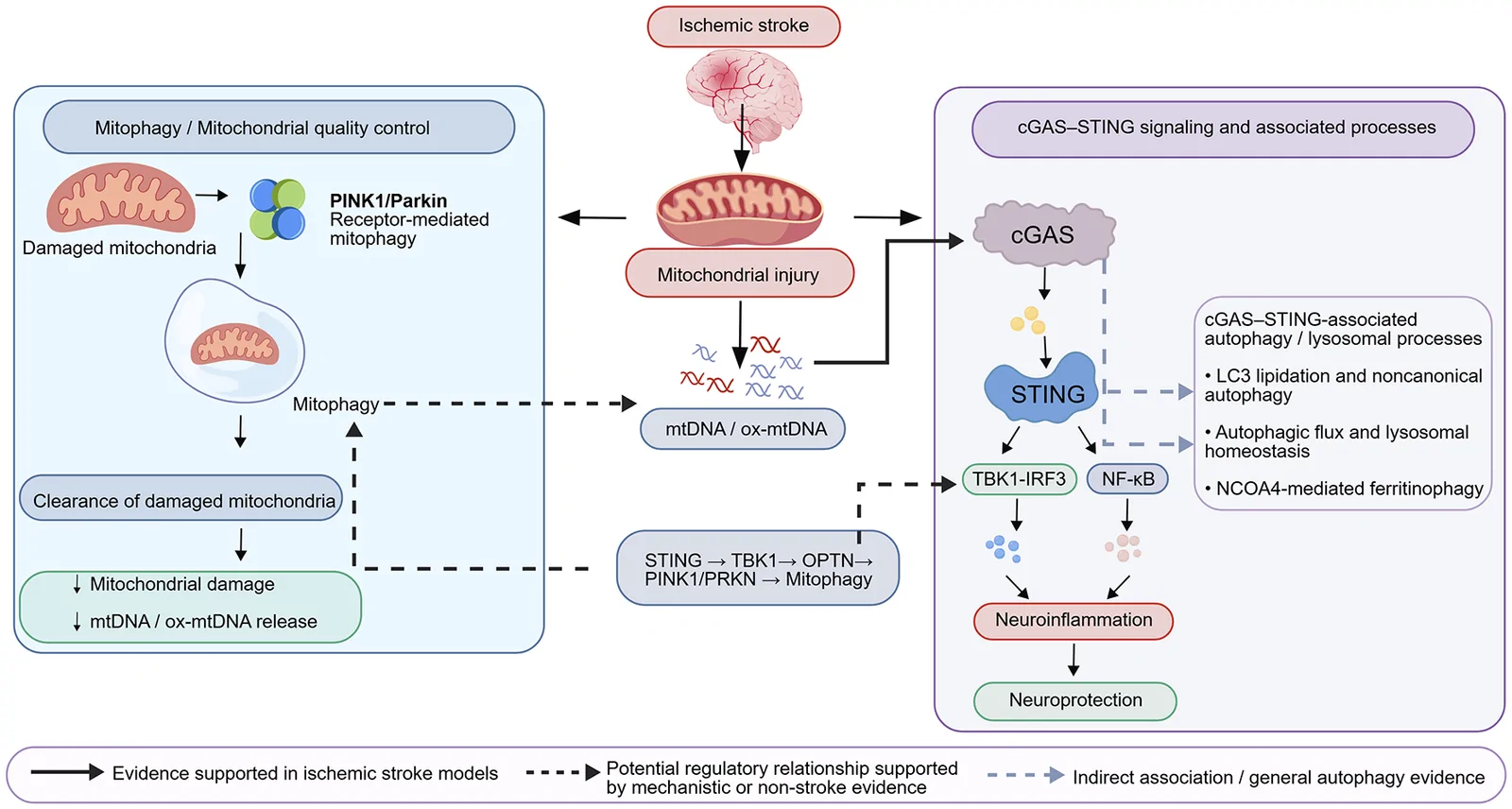
Proposed model of functional synergy and potential molecular crosstalk between mitophagy and the cGAS—STING pathway following ischemic stroke. Mitochondrial injury can release mtDNA/ox-mtDNA and activate cGAS—STING signaling, whereas mitophagy may contribute to damaged mitochondrial clearance and potentially limit mtDNA release. The STING—TBK1—OPTN—PINK1/PRKN axis represents a potential link between cGAS—STING signaling and mitophagy that remains insufficiently validated in ischemic stroke. Solid arrows indicate relationships supported by ischemic stroke models, black dashed arrows indicate potential regulatory relationships supported mainly by mechanistic or non-stroke evidence, and light-blue dashed arrows indicate indirect associations or evidence related to general autophagy. Created with BioGDP.com.

## 5 Therapeutic potential

### 5.1 Therapeutic agents targeting post-stroke cGAS–STING, mitophagy, and their crosstalk

Recent studies have demonstrated that the cGAS–STING pathway and mitophagy not only independently mediate post-stroke neuroinflammation through distinct mechanisms, but may also jointly hold important clinical value in the treatment of ischemic stroke. Accordingly, extensive research has focused on these processes, suggesting that interventions targeting cGAS–STING signaling and mitophagy may represent promising strategies for the prevention and treatment of ischemia-related central nervous system disorders. Representative pharmacological agents are summarized in Table 1.

**Table 1:** Research results of different drugs.

Target/pathway	Drug	Experimental model	Mechanism of action	Key findings/outcomes	References
Direct focal ischemic-stroke					
PINK1/Parkin	Glycosides of Buyang Huanwu decoction	Rat (MCAO/R^a^); primary neural cells (OGD/R^b^)	Activation PINK1/Parkin-mediated mitophagy suppresses inflammation	•Reduced neurological deficit scores and cerebral infarct volume •Reduced neuronal damage and pyroptosis •Improved cellular survival and morphology •Neuroprotective effects were attenuated by mitophagy blockade	Jiao et al., 2024
PINK1/Parkin	Ginsenoside Rg1	Rat (MCAO/R); rat microglia (OGD/R)	Activation PINK1/Parkin-mediated mitophagy suppresses NLRP3 inflammasome activation	•Reduced cerebral infarct area •Reduced neuronal damage and apoptosis •Reduced ROS and inflammatory responses •Improved pathological changes associated with cerebral I/R injury	Sui et al., 2024
PINK1/Parkin	Esculin	Mouse (MCAO/R)	Activation of PINK1/Parkin-mediated mitophagy suppresses NLRP3 inflammasome activation	•Ameliorated neurological deficits and restored cerebral blood flow in MCAO/R mouse •Reduced cerebral infarct size •Preserved neuronal structure and alleviated neuronal damage •Attenuated oxidative stress	Lin et al., 2026
Ox-mtDNA-cGAS–STING axis	Cyclosporine A	Rat (tMCAO/R^c^); HT22^d^ (OGD/R)	Inhibits mitochondrial permeability transition and reduces ox-mtDNA release and subsequent cGAS–STING activation	•Improved neurological deficits •Reduced infarct size •Preserved neuronal structure •Reduced oxidative stress and restored mitochondrial function	Li et al., 2024
NETs -cGAS–STING	DNase I	Mouse (Photothrombotic MCAO); HBMEC^e^ (OGD/R)	Degrades NETs and suppresses NET-associated cGAS–STING/type I IFN signaling	•Reduced BBB disruption •Reduced tPA-associated cerebral hemorrhage •Improved neurological/motor function •Reduced inflammatory signaling associated with NETs	Wang et al., 2021
STING	C-176	Mouse (MCAO/R); HT22 and BV2 microglia (OGD/R)	Blocks STING palmitoylation at Cys91 and suppresses downstream IRF3/NF-κB signaling	•Reduced cerebral infarction and brain edema •Reduced neuronal injury and degeneration •Reduced neuronal apoptosis •Improved long-term neurological and cognitive function	Kong et al., 2022
STING	Edaravone dexborneol	Rat (MCAO/R); microglia (OGD/R)	Inhibits STING, restores STX17-mediated autophagic flux, and suppresses NLRP3 inflammasome activation	•Reduced infarct volume and neuronal damage •Reduced neuroinflammation and pyroptosis-related injury •Preserved mitochondrial function •Promoted neurological recovery	Zhang et al., 2025
cGAS	Melatonin	Mouse (MCAO/R); BV2 microglia (OGD/R)	Suppresses cGAS-STING-associated inflammatory signaling and microglial pyroptosis	•Reduced infarct volume •Reduced neurological deficits •Reduced microglial activation and inflammatory cytokine production •Reduced microglial pyroptosis and peripheral immune-cell infiltration	Li Q. et al., 2026
cGAS	A151	Mouse (MCAO); primary microglia	Competitively interferes with cytosolic DNA sensing and suppresses cGAS/AIM2-associated inflammatory signaling	•Reduced infarct volume and brain edema •Reduced neurobehavioral deficits •Reduced inflammatory-cell infiltration and cytokine production •Reduced cell death and neuronal injury •Improved long-term neurological recovery	Li et al., 2020
STING	H-151	Mouse (photothrombotic stroke model)	Inhibits STING signaling and suppresses STING-associated microglial activation and synaptic phagocytosis	•Reduced microglial synaptic engulfment •Preserved synaptic density •Reduced microglial activation •Improved motor and sensorimotor functional recovery	Wu C. et al., 2024
SIRT3-cGAS–STING	2-APQC	Rat (MCAO/R); primary astrocytes (OGD/R)	Activates SIRT3 and suppresses cGAS–STING inflammatory signaling	•Reduced cerebral infarct volume •Improved neurological function •Reduced astrocytic inflammatory responses •Reduced neuronal apoptosis	Lei et al., 2026
cGAS–STING	Geniposide	Mouse (MCAO/R); SH-SY5Y^f^ (OGD/R)	Inhibits excessive cGAS–STING activation and associated excessive neuronal autophagy	•Reduced cerebral infarct volume •Reduced neuronal apoptosis •Improved neuronal viability •Improved neurological function	Fuyun et al., 2025
cGAS	RU.521	Mouse (MCAO/R)	Inhibits cGAS signaling and reduces cGAS-associated NCOA4 ferritinophagy	•Reduced cerebral infarct volume •Reduced oxidative stress •Reduced excessive ferritinophagy/autophagy and apoptosis •Alleviated early cerebral I/R injury	Li B. et al., 2023
cGAS–STING	4-sulfonic calixarenes	Mouse (tMCAO)	Primarily inhibits AIM2 by competitively blocking dsDNA binding; at higher concentrations also inhibits cGAS and TLR9	•Reduced AIM2-dependent post-stroke T-cell death •Attenuated post-stroke immunosuppression •Suppressed DNA-driven inflammatory signaling	Green et al., 2023
15.6-7.4,-38.8498ptcGAS	Activin A	Mouse (MCAO/R); primary cortical neurons (OGD/R)	Inhibits cGAS–STING-mediated excessive autophagy through PI3K–PKB signaling	•Increased neuronal survival •Reduced infarct size •Reduced ischemic neuronal injury •Improved neurological function	Liu et al., 2023
Related CNS ischemic/hypoxic					
Miro1-PINK1	Dihuang Yinzi	Rat (post-stroke depression model)	Promotes Miro1 degradation, enhances PINK1-mediated mitophagy, and suppresses NF-κB-mediated inflammation	•Reduced depressive-like behaviors •Reduced cerebral infarct volume •Reduced neuroinflammatory responses •Therapeutic effects were reversed by mitophagy inhibition or Miro1 overexpression	Chang et al., 2026
Lysosome/autophagy–mitophagy	URB597	Rat (bilateral common carotid artery occlusion model)	Restores impaired autophagy/mitophagy and lysosomal function, thereby suppressing NLRP3–CASP1 activation	•Reduced microglial overactivation and ROS accumulation •Reduced NLRP3/CASP1 inflammatory signaling •Reduced pro-inflammatory cytokine production •Alleviated chronic cerebral ischemic injury	Su et al., 2019
Mitochondrial protection	Glycosylated colchicine derivative 116B	Rat (tBCCAO/R^g^ stroke model); SH-SY5Y (oxidative stress model)	Restores mitochondrial function, inhibits MAPK-driven inflammation and decreases microglia and astrocyte activation	•Reduced infarct volume •Improved neurological scores	Jash et al., 2026
cGAS–STING pathway	Hydrogen sulfide	Mouse (post-stroke depression); BV2 microglia (OGD)	Inhibits cGAS–STING signaling and enhances ER-phagy	•Reduced depressive-like behaviors •Reduced hippocampal injury •Reduced pro-inflammatory markers and microglial inflammatory responses	Luo et al., 2025
DNA-cGAS–STING axis	Artesunate	Rat (bilateral common carotid artery occlusion model); *in vitro* model (OGD)	Activates lysosomal function and suppresses cGAS—STING-mediated inflammatory signaling	•Reduced neuronal injury and cell death •Improved learning and memory •Reduced chronic ischemic brain injury	Wei et al., 2025
15.6-7.4,-51.5498ptSIRT3-mtDNA-cGAS–STING	(+)-JQ1	Rat (hypoxic-ischemic encephalopathy model); HT22 (OGD/R)	Upregulates SIRT3, preserves mitochondrial homeostasis, reduces mtDNA release, and suppresses cGAS–STING signaling	•Reduced cerebral infarction and edema •Reduced neuronal loss •Improved emotional and cognitive function •Reduced neuroinflammation and ferroptosis	Yu et al., 2026
Supportive non-cerebral I/R					
Oxidative-stress modulation	Mitochondria targeting traizine base antioxidant	Rat; *vitro* model	Inhibits mitoautophagy and decreases neuroinflammation	•Mitigated LPS-triggered oxidative stress •Exhibited stronger neuroprotective effects than aspirin	Garg et al., 2025
cGAS	RU.521	Mouse (cerebral venous sinus thrombosis model)	Inhibits cGAS activity and downstream STING-associated inflammatory signaling	•Reduced neuroinflammation and oxidative stress •Reduced NLRP3 inflammasome activation and pyroptosis •Reduced neuronal apoptosis and degeneration •Improved motor/neurological function	Ding et al., 2022

^a^MCAO/R, middle cerebral artery occlusion/reperfusion.

^b^OGD/R, oxygen-glucose deprivation/reperfusion.

^c^MCAO/R, transient middle cerebral artery occlusion/reperfusion.

^d^HT22, immortalized mouse hippocampal neuronal cell.

^e^HBMEC, human brain microvascular endothelial cells.

^f^SH-SY5Y, human neuroblastoma cell line.

^g^tBCCAO/R, transient bilateral common carotid artery occlusion reperfusion.

With continuous advances in therapeutic strategies driven by global scientific research, gene-based interventions and molecular biology tools, in addition to pharmacological approaches, have also played an important role in the treatment of ischemic stroke (Table 2). Although small-molecule cGAS–STING inhibitors have demonstrated strong anti-inflammatory effects *in vitro* and in animal models, their clinical translation for ischemic stroke remains challenging. First, most existing small-molecule inhibitors of cGAS–STING exhibit limited ability to cross the blood–brain barrier (BBB), making it difficult to achieve therapeutically effective concentrations in the brain (Liu et al., 2023). Second, the cGAS–STING pathway is involved not only in sterile inflammation but also in critical host antiviral and antibacterial immune defenses; therefore, systemic administration of cGAS–STING inhibitors must be approached with caution (Liu et al., 2023). To address these limitations, future drug development should focus on intelligent nanodelivery systems responsive to the ischemic microenvironment. Such systems can achieve targeted accumulation in ischemia/reperfusion lesions through biomimetic modifications while minimizing excessive interference with systemic innate immunity, thereby expanding the translational potential of cGAS–STING modulators in clinical applications.

**Table 2:** Gene interventions and molecular biology tools.

Target/pathway	Treatment	Experimental model	Mechanism of action	Key findings/outcomes	References
Direct focal ischemic stroke					
HIF-1α- BNIP3	HIF-1α overexpression plasmids	Mouse (MCAO/R); SH-SY5Y (OGD/R)	Activates HIF-1α-BNIP3-dependent protective mitophagy and suppresses NLRP3 inflammasome activation	•Reduced cerebral infarct volume •Reduced neurological deficits •Reduced neuronal injury/apoptosis •Attenuated NLRP3-associated inflammation	Huo et al., 2026
ATF4-Parkin	AAV^a^-mediated ATF4 overexpression	Rat (MCAO/R)	Upregulates Parkin-dependent mitophagy and suppresses NLRP3 inflammasome activation	•Reduced cerebral infarct volume •Improved neurological scores •Improved histopathological neuronal injury •Reduced inflammatory responses	He et al., 2019
PINK1/Parkin	LncRNA Tug1 knockdown	Mouse (photothrombotic stroke model); BV2 microglia (OGD/R)	Tug1 knockdown enhances PINK1/Parkin-mediated mitophagy and suppresses microglial pyroptosis	•Reduced microglial pyroptosis •Reduced neuronal damage •Improved neurological outcome •Protective effects were reversed by PINK1 silencing or mitophagy inhibition	Yao et al., 2025
PGC-1α- ULK1	PGC-1α upregulation	Mouse (tMCAO/R)	Promotes ULK1-dependent autophagy/mitophagy and suppresses NLRP3 activation	•Reduced neurological deficits •Reduced NLRP3 activation and pro-inflammatory cytokines •Enhanced mitochondrial clearance and autolysosome formation •Neuroprotection was abolished by ULK1 inhibition/knockdown	Han et al., 2021
cGAS	FTO overexpression	Mouse (MCAO/R); primary isolated microglia and BV2 microglia (OGD/R)	Reduces cGAS mRNA stability through m6A demethylation and suppresses cGAS–STING/NF-κB signaling	•Reduced microglia-mediated inflammatory responses •Alleviated cerebral I/R-associated brain injury •Reduced inflammatory signaling associated with cGAS–STING	Yu et al., 2023
15.6-7.4,-63.7498ptcGAS	cGAS knockdown	Mouse MCAO; BV2/primary microglia stimulated with OGD-induced DAMPs	Suppresses cGAS–STING signaling and shifts microglia away from the pro-inflammatory phenotype	•Reduced microglial M1 polarization •Reduced inflammatory responses •Reduced brain-cell apoptosis •Reduced cerebral infarction •Improved neurological function	Jiang et al., 2021
Related CNS ischemic/hypoxic					
Mitophagy–cGAS–STING	RORα overexpression	Rat (hypoxic-ischemic encephalopathy model); BV2 microglia (OGD/R)	Activates mitophagy, reduces mtDNA leakage, and suppresses the mtDNA–cGAS–STING–NLRP3 axis	•Reduced mtDNA leakage and ROS accumulation •Reduced IL-1β and NLRP3-associated inflammation •Improved cognitive and motor function •Neuroprotective effects were reversed by mitophagy inhibition	Song et al., 2025
cGAS/STING–LINE-1	STING siRNA/LINE-1 inhibition	Rat (hypoxic-ischemic encephalopathy model)	STING inhibition reduces lysosome-associated Cathepsin B and apoptotic signaling; LINE-1 may act upstream of cGAS/STING	•Reduced infarct area •Reduced cortical neuronal degeneration •Reduced Bax expression and caspase-3 cleavage •Reduced neurological impairment	Gamdzyk et al., 2020

^a^AAV, Adeno-associated virus.

### 5.2 Application of nanomaterials in alleviating post-stroke inflammation

In recent years, nanomaterial-based strategies targeting key pathological processes involved in post-ischemic stroke neuroinflammation have emerged as promising therapeutic approaches. The design and functionalization of nanomaterials provide a versatile platform for modulating inflammatory signaling pathways, restoring mitochondrial homeostasis, and regulating the ischemic microenvironment. Compared with conventional small-molecule inhibitors, nanoplatforms may offer advantages in improving stability, targeting efficiency, and blood–brain barrier (BBB) penetration. Through surface modification with targeting ligands or functional molecules, nanoparticles can also achieve more precise delivery to specific cells or tissues (Hu and Tian, 2024). Among these strategies, targeting the cGAS–STING pathway and restoring mitochondrial homeostasis have attracted increasing attention because of their important roles in post-stroke neuroinflammation. However, due to the complexity of the post-stroke immune microenvironment, blockade of a single pathway is often insufficient to achieve optimal therapeutic efficacy. Accordingly, an increasing number of studies have focused on developing multifunctional intelligent nanoplatforms to regulate this pathway through multidimensional synergistic mechanisms precisely.

One key strategy for suppressing excessive cGAS–STING activation is to prevent mtDNA release at its source. In this regard, Chen et al. (2026) developed natural organic-derived carbon dots (nCDs) with superoxide dismutase (SOD)- and catalase (CAT)-like activities. This system maintains mitochondrial metabolic homeostasis by precisely targeting key IDH1 sites. As a result, it effectively reduces cytosolic mtDNA leakage, blocks subsequent cGAS–STING activation, and inhibits microglial polarization toward a pro-inflammatory phenotype. It also exerts neuroprotective effects in both zebrafish and mouse models. However, the advantages of nCDs are also accompanied by potential limitations. Although no obvious nCDs-related toxicity has been observed in current studies, their biological effects may not be entirely restricted to the currently identified IDH1-dependent regulatory axis. Global redox-omics analysis revealed that nCDs treatment induced significant redox alterations in 104 proteins, suggesting that nCDs may trigger broader molecular responses. Similarly, Xiong et al. (2026) constructed a strontium (Sr)-substituted Prussian blue analog nanodrug (SrHCF). This system integrates ROS scavenging, ferroptosis inhibition, and calcium antagonism. By preserving mitochondrial integrity, it suppresses the upstream cGAS–STING pathway, significantly reduces neuronal death, and promotes functional recovery. Although SrHCF can simultaneously modulate oxidative stress, ferroptosis, calcium overload, and inflammation, this multifunctionality also complicates the attribution of individual therapeutic effects to specific mechanisms and may potentially lead to unpredictable off-target effects. Furthermore, because the activity of SrHCF involves Fe^2+^-responsive structural transformation and metal ion release, the degradation of its inorganic components requires further investigation (Feliu et al., 2016). For free DNA that has already been released into the cytosol, conventional enzyme-based therapy is limited by high cost and poor stability. This highlights the need for alternative strategies. Zhu et al. (2023) addressed this challenge by designing a DNase-mimicking artificial enzyme loaded with the STING inhibitor C-176. This system achieves a synergistic effect of “upstream clearance” and “downstream inhibition.” Its nuclease component efficiently degrades intracellularly accumulated mtDNA, while the released C-176 directly blocks downstream STING signaling, thereby protecting neurons in the ischemic penumbra. Combining DNA degradation with STING inhibition provides a powerful strategy for suppressing innate immune activation; however, excessive interference with cytosolic DNA-sensing systems may potentially affect physiological immune functions (Hopfner and Hornung, 2020; Ge and Zhang, 2023). Beyond directly targeting free DNA and STING, regulating the complex immune cell network within ischemic lesions is another important approach for enhancing anti-inflammatory efficacy. Sun et al. (2023) identified a pathogenic interaction between NETs and the cGAS–STING pathway and developed an intelligent liposomal system accordingly. This system simultaneously inhibits NET release and cGAS–STING activation, thereby providing dual protection for the blood–brain barrier (BBB) and neurons. However, the targeting specificity of this system depends on ROS release, whereas oxidative stress is also present in other inflammatory conditions, potentially resulting in additional off-target effects. Moreover, prolonged or systemic inhibition of NETs may interfere with physiological antimicrobial defense (Brinkmann et al., 2004). In addition, Shi et al. (2022) designed an engineered biomimetic immunosuppressive nanoparticle (VIN). This nanoplatform enables the local release of the cGAS inhibitor A151 within the central nervous system, promoting microglial polarization toward an anti-inflammatory M2 phenotype. At the same time, its functionalized surface adsorbs and neutralizes the chemokine CXCL12, thereby inhibiting the infiltration of peripheral immune cells into the brain. These combined actions significantly reduce infarct volume and mortality. Nevertheless, CXCL12 neutralization may require careful consideration because CXCL12 also participates in vascular remodeling and tissue repair (Nassari et al., 2017; Janssens et al., 2018). In addition, mesenchymal stem cells (MSC) membrane-derived nanoplatforms face challenges related to membrane source variability, preservation of functional proteins, and reproducibility of biological properties (Lopes et al., 2022; Pittenger et al., 2019; Zou et al., 2023).

Beyond direct cGAS-STING targeting, emerging mitochondria-targeted nanomaterials have also demonstrated therapeutic potential for alleviating post-stroke neuroinflammation. Tang et al. developed a biomimetic PP@PCL nanoplatform by fusing 4T1 tumor-cell membrane with platelet membrane onto paeonol- and polymetformin-loaded liposomes. This design combined BBB penetration and ischemic-lesion targeting with ROS scavenging, neuroinflammatory modulation, microglial reprogramming, and angiogenic effects, thereby addressing multiple pathological processes of ischemic stroke within a single platform (Tang et al., 2024). However, the use of tumor-cell membranes raises additional concerns regarding membrane composition, antigenic components, and long-term biosafety (Ye et al., 2024; He et al., 2024). Moreover, the simultaneous integration of BBB penetration, lesion targeting, ROS scavenging, immune modulation, and angiogenic regulation may increase the risk of unpredictable interactions. Therefore, it is important to determine whether each mechanism acts independently. Zhang et al. developed a mitochondrial-targeted and ROS-responsive nanoparticle system (SPNPs) containing puerarin and decorated with the mitochondrial-targeting peptide SS-31. Encapsulation in a thermosensitive hydrogel enabled intranasal administration and nose-to-brain delivery, thereby bypassing the BBB while promoting accumulation in the ischemic penumbra. This strategy simultaneously addressed oxidative stress and mitochondrial dysfunction and reduced neuroinflammation and neuronal apoptosis in MCAO models (Zhang et al., 2023). However, the nose-to-brain route does not completely eliminate delivery barriers. Nasal mucociliary clearance and interindividual variability in nasal absorption remain important challenges (Gao et al., 2026). Yin et al. recently developed a bioengineered nanolamellar system (MM@BPPF) consisting of a microglia–mitochondria hybrid membrane, black phosphorus nanosheets, polymetformin, and fingolimod. This platform integrates inflammation-directed targeting, mitochondrial homotypic targeting, ROS scavenging, sequential drug release, and microglial immunomodulation. Importantly, intranasal administration bypassed the BBB and enhanced accumulation in ischemic brain tissue and mitochondria, providing a highly integrated strategy for simultaneously restoring mitochondrial homeostasis and controlling neuroinflammation (Yin et al., 2026). However, this bioengineered nanolamellar system consists of a hybrid nanovesicle composed of two membrane components and is loaded with two therapeutic agents. Therefore, the biocompatibility of the membrane materials requires further evaluation (Feng et al., 2025). For example, it is important to ensure that allogeneic or xenogeneic membrane components do not induce adverse immune responses (Feng et al., 2025). In addition, black phosphorus can degrade into phosphate in the body, while the substantial ROS generated by its photothermal effects may be toxic to DNA in normal cells, potentially causing progressive oxidative damage and ultimately cell death (Li H. X. et al., 2023). Therefore, determining an appropriate dose and assessing whether long-term use may lead to excessive phosphate accumulation and toxicity are critical.

These studies demonstrate the strong potential of nanomaterials for targeted Post-Stroke Inflammation. Systematic optimization of nanomaterial design may help overcome current limitations in delivery efficiency and targeting specificity, thereby improving therapeutic outcomes in ischemic stroke. Although encouraging results have been obtained in animal models, further studies are still needed to clarify their safety, efficacy, and translational feasibility in clinical settings. In addition, the translation of nanoplatforms from laboratory research to clinical applications faces numerous challenges, including the feasibility of large-scale manufacturing, cost-effectiveness, batch-to-batch consistency, long-term stability and safety, and regulatory evaluation. Particularly for cell membrane-based biomimetic nanoplatforms, several major challenges remain: allogeneic membranes may exhibit immunogenicity and potentially elicit immune responses following repeated administration. Meanwhile, variations in time points, age, diet, medication use, and circadian rhythms may alter membrane protein expression, thereby reducing the reproducibility of formulation preparation (Feng et al., 2025). Despite these technical challenges, ongoing progress in research and drug development suggests that nanomaterial-based strategies targeting the cGAS–STING pathway hold considerable promise for the treatment of ischemic stroke.

## 6 Perspectives

In the in-depth investigation of post-stroke neuroinflammation, the synergistic crosstalk between mitophagy and the cGAS–STING pathway presents both challenges and promising therapeutic opportunities. Although the dual roles of mitophagy in ischemic stroke have been well-established, its therapeutic window remains unclear and is likely context dependent (Wu J. et al., 2025; Wu Y. et al., 2025). This makes it difficult to define the precise threshold at which mitophagy shifts from a protective mechanism to a pathological process, thereby complicating the development of targeted interventions. In addition, the specific mechanisms by which mitophagy regulates neuroinflammation, such as its effects on microglial polarization, still require more comprehensive clarification. Beyond these intracellular processes, the integrity of the neurovascular unit (NVU) must also be considered. Intercellular communication plays a crucial role in this framework. For example, cGAS–STING-mediated inflammatory chemokine signaling within the NVU can further activate microglia and astrocytes, thereby amplifying the neuroinflammatory response. Moreover, blocking gap junction (GJ)-mediated signaling has been shown to reduce cGAS–STING activation and inhibit the spread of neuroinflammation (Elzinga et al., 2022). Therefore, future immunotherapeutic strategies should regard the NVU as an integrated functional unit rather than focusing solely on microglia. At the same time, the molecular crosstalk between mitophagy and cGAS–STING still requires deeper investigation in the brain. Although modulation of mitophagy to influence the cGAS–STING pathway has been shown to improve inflammatory responses in ischemia/reperfusion injury of other organs, related studies in cerebral ischemia/reperfusion remain limited (Zhou et al., 2026; Li Y. et al., 2026).

Current evidence supports the view that the functional synergy and potential molecular crosstalk between mitophagy and the cGAS–STING pathway are closely associated with the progression of neuroinflammation after ischemic stroke. Since these findings are mainly derived from animal models, further clarification of these mechanistic relationships and their therapeutic relevance, followed by clinical validation, is warranted. Such efforts may reveal new therapeutic targets and promote the development of integrated treatment strategies for ischemic stroke.
